# Identification of novel regulators in T-cell differentiation of aplastic anemia patients

**DOI:** 10.1186/1471-2164-7-263

**Published:** 2006-10-19

**Authors:** Anke Franzke, Robert Geffers, J Katrin Hunger, Susanne Pförtner, Wenji Piao, Philipp Ivanyi, Jens Grosse, Michael Probst-Kepper, Arnold Ganser, Jan Buer

**Affiliations:** 1 Department of Hematology, Hemostasis and Oncology, Hannover Medical School, Carl-Neuberg-Str.1, D-30625 Hannover, Germany; 2 Department of Cell Biology and Immunology, Helmholtz Centre for Infection Research, Inhoffenstraße 7, D-38124 Braunschweig, Germany; 3 Department of Medical Microbiology, Hannover Medical School, Carl-Neuberg-Str.1, D-30625 Hannover, Germany; 4 Junior Research Group for Xenotransplantation, Department of Visceral and Transplant Surgery, Hannover Medical School, Carl-Neuberg-Str.1, D-30625 Hannover, Germany

## Abstract

**Background:**

Aplastic anemia (AA) is a bone marrow failure syndrome mostly characterized by an immune-mediated destruction of marrow hematopoietic progenitor/stem cells. The resulting hypocellularity limits a detailed analysis of the cellular immune response. To overcome this technical problem we performed a microarray analysis of CD3^+ ^T-cells derived from bone marrow aspirates and peripheral blood samples of newly diagnosed AA patients and healthy volunteers. Two AA patients were additionally analyzed after achieving a partial remission following immunosuppression. The regulation of selected candidate genes was confirmed by real-time RT-PCR.

**Results:**

Among more than 22.200 transcripts, 583 genes were differentially expressed in the bone marrow of AA patients compared to healthy controls. Dysregulated genes are involved in T-cell mediated cytotoxicity, immune response of Th1 differentiated T-cells, and major regulators of immune function. In hematological remission the expression levels of several candidate genes tend to normalize, such as immune regulators and genes involved in proinflammatory immune response.

**Conclusion:**

Our study suggests a pivotal role of Th1/Tc1 differentiated T-cells in immune-mediated marrow destruction of AA patients. Most importantly, immune regulatory genes could be identified, which are likely involved in the recovery of hematopoiesis and may help to design new therapeutic strategies in bone marrow failure syndromes.

## Background

Acquired aplastic anemia (AA) is a rare bone marrow failure state characterized by marrow hypocellularity and low peripheral blood cell counts [1]. Although the exact pathogenic mechanism is still unknown, beside a primary stem cell defect [2] and a disturbed microenvironment [3], pre-/clinical data suggest an immune-mediated destruction of marrow progenitor/stem cells [1,4]. Similar to other autoimmune diseases, antigen-specific T cells could be expanded from the bone marrow of AA patients and likely mediate organ-specific cytotoxicity to hematopoietic stem cells and progenitor cells [5]. In addition, it has been reported that the bone marrow of AA patients contains increased numbers of apoptotic cells [6] and that hematopoietic stem cells of AA patients express Fas antigen [7]. Peripheral blood and bone marrow T-cells show signs of activation [8], secrete high levels of interferon-gamma (IFN-γ) and tumor-necrosis factor alpha (TNF-α). These two cytokines may suppress the proliferation of early and late hematopoietic progenitor cells and initiate apoptosis by induction of Fas on CD34^+ ^stem cells [9,10]. Most recently, some groups reported a polarization of CD4^+ ^T-cells towards a type-1 immune response that leads to activation of cytotoxic CD8^+ ^T-cells with destruction of marrow stem cells [11]. However, genes important for the recruitment of effector T-cells to the bone marrow as target organ of autoimmunity and regulatory molecules directing their differentiation have not been identified so far. Therefore, we performed a microarray study comparing gene expression profiles of circulating and marrow-derived T-cells at initial presentation and after hematological recovery in order to elucidate more details of the mechanism resulting in severe bone marrow failure.

## Results

### Microarray analysis

Comparative gene expression profiling was performed in severe aplastic anemia (SAA) patients and healthy controls by analyzing pooled CD3+ T-cells isolated from peripheral blood (PB) and bone marrow (BM) samples (Table 1). Each oligonucleotide microarray (Affymetrix HG_U133A) contained 22.218 human probesets and in our experiments discovered between 48.1% and 53.7% "present calls" as calculated by the statistical detection algorithm of Affymetrix. In general, "present calls" of approximately 50% provide confidential results. Scaled factors which were used to normalize the arrays to an average intensity ranged from 0.915 to 1.544 showing the high reproducibility of the experimental procedure. Furthermore, the hybridisation was monitored by the usage of hybridisation controls comparing total RNA amount and final signal intensity on the array (r > 0.99). Taken together, our data reached a highly acceptable quality level for further analysis.

**Figure 1:**
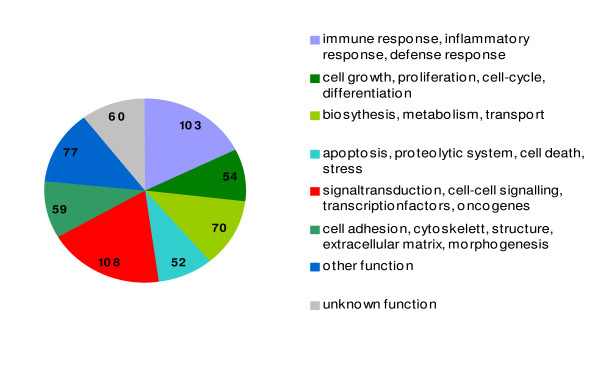
**Results of differential transcriptome analysis in bone marrow-derived CD3+ T-cells of SAA patients**. Genome-wide gene expression profiles of CD3+ T-cells derived from bone marrow aspirations of SAA patients and healthy volunteers were comparatively analyzed. The diagram summarizes the number of differentially expressed genes (> 2-fold) assigned to different functional classes.

To eliminate genes which may be individually regulated independent of disease we focused in our study only on genes coregulated in both of the analyzed SAA pools (pool I and IIa) in comparison to the normal controls (pool III), each separately for PB and BM samples. The potentially disease-specific (dys-)regulation comprised a wide variety of genes belonging to different functional classes, such as immune response, proliferation/cell growth, biosynthesis/metabolism, signal transduction and apoptosis (Figure 1). The most interesting genes are summarized below, whereas the entire data set is available at the GEO database [12] under the accession number GSE3807.

### Gene expression profiles of T-cells from SAA patients at initial diagnosis

The circulating T-cells isolated from the PB samples of SAA patients exhibited a broadly normal gene expression profile with dysregulation of only 42 genes (35 up- and 7 downregulated) in both of the analyzed SAA pools. In contrast, in comparison to the control pool T-cells isolated from BM aspirations showed a dysregulation of 583 genes (125 up- and 458 downregulated), which were coregulated in both SAA pools. Among the regulated genes identified in circulating T-cells some were also differentially expressed in BM-derived T-lymphocytes (i.e. IRS2, BASP1, EGR1, Lyn) and other molecules were only regulated in PB samples (i.e. IL6R, TNFAIP3, BCL3). Differentially expressed genes, which were exclusively found in BM-infiltrating T-cells, were classified into several functional categories (Figure 1). These genes included molecules involved in immune responses and their regulation with the following fold changes for SAA pool I and IIa, respectively: PF-4 (-157-fold; -188-fold), CD26 (+2.2-fold; +2.1-fold), Ncf-1 (-3.9-fold; -2.2-fold), CCR2 (+2.7-fold; +2.7-fold), CX3CR1 (+4.3-fold; +2.9-fold) and other chemokine receptors and ligands. Many cytokines/growth factors and their receptors, which play an important role in inflammation and endogenous response to infections, were found to be dysregulated like IL-1α (-25.9-fold; -40.3-fold), IRAK3 (-5.9; -88-fold), LTβ (+2.2-fold; +2.2-fold), Toll-like receptor 2 (-10.4-fold; -5.0-fold) and CIAS1 (-23.5-fold; -6.3-fold). Furthermore molecules important for the regulation of proliferation and cell cycling were dysregulated (i.e., SOCS2, JAG1, VEGF, SKIL). Finally, several transcription factors were found to be differentially expressed in SAA patients (i.e., STAT1, STAT5B and IRF2).

To obtain more information, we also set a minimum fold change of 1.5 revealing an upregulation of genes in BM-derived CD3+ T-cells from SAA patients playing an important role in the regulation of apoptosis, like TRADD (+1.8-fold; +1.8-fold), TRAF5 (+1.8-fold; +1.8-fold), perforin-1 (+2.2-fold; +1.7-fold), granzyme H (+2.9-fold; +1.8-fold) and granzyme B (+2.6-fold; +1.5-fold).

**Figure 2:**
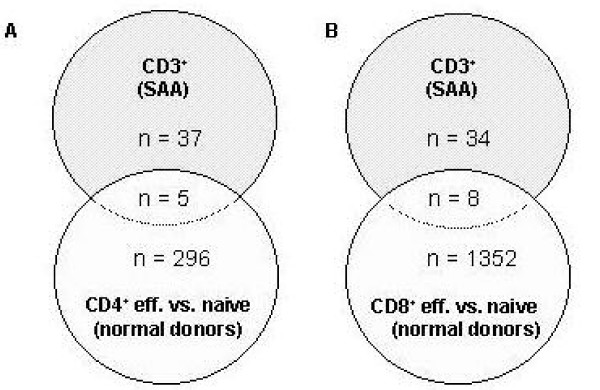
**Gene expression profile analysis of normal T-cell subpopulations in comparison to differentially expressed genes in CD3+ T-cells of SAA patients**. The gene expression data of effector memory T-cell populations were compared separately for each of 3 normal donors with the mean gene expression level of the respective naive T-cell populations [13, 14]. Significantly regulated genes (p < 0.05, student's t-test) were further analyzed in comparison to the differential gene expression pattern of SAA patients (pool I and pool IIa of the PB). Number of commonly and differentially expressed genes is shown for effector memory versus naïve CD4+ T-cells in comparison to circulating CD3+ T-cells of SAA patients (part A) and also for the respective CD8+ T-cell subpopulations (part B).

In order to examine whether the obtained differential gene expression profiles in the analyzed SAA patients simply reflect an over- or under-representation of T-cell subpopulations, the differential gene expression pattern identified in circulating T-cells of SAA patients (pool I and IIa of the PB) were compared with gene expression profiles of effector memory versus naive CD4^+ ^and CD8^+ ^T-cell populations generated from normal donors [13,14]. The comparative analysis of effector memory and naïve CD4^+ ^(effector memory and naïve CD8^+ ^T-cells, respectively) shows that 301 genes (n = 1360, respectively) are statistically singificantly regulated in one of the analyzed subpopulations (p < 0.05, student's t-test). Interestingly, only 5 (n = 8, respectively) of these genes exhibited a similar expression pattern in the circulating CD3+ T-cells of the SAA patient pools (Figure 2).

### Gene expression profiles of CD3^+ ^T-cells from AA patients in remission

**Figure 3:**
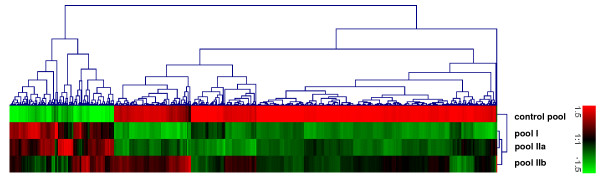
**Gene expression profiles of bone marrow-derived CD3+ T-cells of SAA patients at initial presentation and after hematological recovery**. Hierarchical clustering analysis of gene expression patterns in CD3+ T-cells of 2 independent SAA patient pools at initial presentation (I and IIa) and in hematological remission (pool IIb) following immunosuppressive therapy were performed with respect to the expression profile of pooled CD3+ T-cells from healthy volunteers.

**Figure 4:**
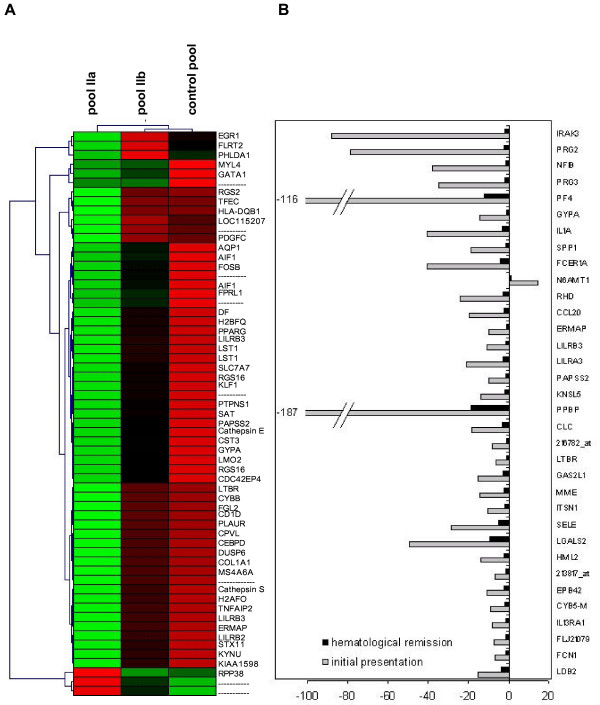
**Changed gene expression pattern following immunotherapy**. Panel A: Hierarchical clustering analysis includes genes that tend to normalize after immunotherapy with respect to their gene expression levels detected in the normal control pools. In the SAA patient pool of bone-marrow derived T-cells 60 of 583 regulated genes turned to nearly normal gene expression levels after successful immunosuppression. Panel B: The comparison of the gene expression profiles from bone marrow-derived T-cells of the SAA pool before and after immunotherapy revealed several genes exhibiting a very high difference (> 4-fold) in their expression level.

Gene expression profiles (Figure 3) were performed of CD3+ T-cells isolated from PB and BM samples of 2 SAA patients after achieving a hematological remission following immunosuppressive therapy (pool IIb). Data were analyzed with statistical tools as described in the method part and directly compared to their respective gene expression profile at initial presentation (pool IIa). In order to provide a better overview, we performed a hierarchical cluster analysis including genes that tend to normalize with respect to the gene expression levels detected in the normal control pools (Figure 4a). In total, we identified 60 of 583 regulated genes in the BM samples of the analyzed SAA patients turning to nearly normal gene expression levels after successful immunotherapy. This category mainly included genes involved in the regulation of immune response like Ncf1. In the PB samples of the SAA pool only one of 42 regulated genes (MT1E) tended to expression levels close to the normal control pool, but its function in the immune system is unknown.

Furthermore, the comparison of the gene expression profiles (pool IIa vs IIb) revealed a group of several genes with a very high difference (> 4-fold) in the expression level before and after successful immunotherapy (Figure 4b). Most of these genes are important during inflammation and regulation of immune response, such as platelet-factor 4 (PF4), proteoglycan 2 und 3 (PRG2, PRG3), IL-1α, IRAK3, osteopontin (SPP1), receptor for Fc fragment of IgE (FCER1A) or RNASE3. The fold changes of regulated candidate genes are summarized in Table 2.

### Validation of microarray data by real-time RT-PCR

**Figure 5:**
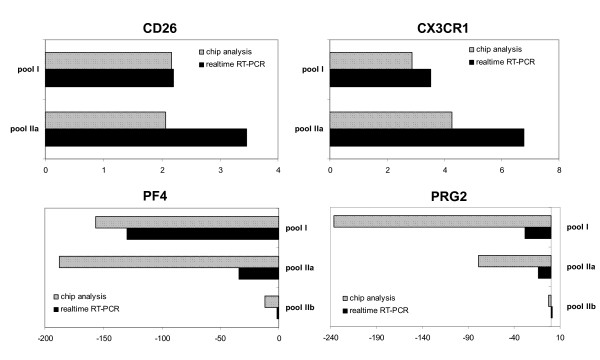
**Validation of microarray results by real-time PCR**. Selected genes (CXCL4, PRG2, CD26, CX3CR1) with differential gene expression in the analyzed SAA and control pools were quantified by Taqman RT-PCR in 2 independent experiments. Relative mRNA expression levels were normalized with respect to RPS9 gene expression as internal control. Results are shown as mean fold-change value of the respective gene expression in the control pool. Slanted bars: Microarray result; black bar: respective real-time RT-PCR result. Panel A shows upregulated genes. Panel B demonstrates downregulated genes.

To confirm the microarray results, quantitative real-time RT-PCR of selected candidate genes was performed using the original patient sample pools (pool I, IIa) and patient 6 in hematological remission. Because of limited amounts of pooled RNA, 4 genes were selected for quantification of gene expression using a new pool of normal BM samples. Even with this new sample pool the regulation of gene expression could be confirmed for the 4 analyzed genes (Figure 5). For example, separate microarray experiments revealed an increased expression for CD26 in pools I and IIa (2.17-fold; 2.07-fold), which could be confirmed by real-time RT-PCR (2.2-fold; 3.5-fold). In general, real-time RT-PCR results correlated well with the differential gene expression data of the microarray experiments.

## Discussion

In acquired aplastic anemia, the evidence of an autoimmune pathogenesis is mostly indirect and the characterization of the underlying immune response is incomplete mainly due to technical difficulties resulting from the disease-specific hypocellularity. However, there is growing evidence that AA results from autoaggressive destruction of hematopoietic stem cells and progenitors mediated by T-cells recognizing inciting target antigens [1]. Several groups have identified clonal T-cell expansion [15-17], proinflammatory cytokine production [9] and T-cell mediated cytotoxicity to CD34+ stem cells [5,7] supporting an antigen-driven T-cell response. Most recently, we could characterize the antigen-binding sites of clonally expanded T-cells in SAA patients and identified the emergence of new T-cell clones after successful immunosuppression [18], suggesting a regulatory T-cell population contributing to the recovery of hematopoiesis. In this study, we applied microarray technology to characterize the immune response of the T-cell system in SAA. The design of our experiments differed from most recently reported transcript profiles of AA patients [19] in the following important issues: (a) The untreated patients included in this study were diagnosed as SAA with similar clinical characteristics, HLA background, response to therapy and without an expanded PNH clone. (b) Gene profiles of CD3+ T-cells from PB and BM samples of the same SAA patients and healthy controls were compared, as the pathological T-cell response is expected in the bone marrow as target organ of AA. (c) Furthermore, the gene profiles of SAA patients were comparatively analyzed with respect to normal gene expression before and after successful immunotherapy in order to identify candidate genes relevant for the recovery of hematopoiesis.

As expected the gene profiles of CD3+ T-cells isolated from PB samples differed significantly from the expression pattern found in BM-derived T-cells of SAA patients. The number of differentially expressed genes in circulating T-cells of SAA patients compared to healthy controls was surprisingly low: Only 42 genes were differentially expressed in peripheral T-cells of the patients pools. Interestingly among these genes, a negative regulator of cytokine response, the zinc finger protein TNFAIP3, was upregulated. The induction of TNFAIP3 may result from cellular counterregulation in response to high serum levels of TNF and IL-1 [20], as this molecule specifically inhibits the respective signal transduction pathways [21]. Two important multifunctional regulators of hematopoiesis, MIP1α [22] and Skil [23], were regulated in both peripheral and marrow-derived T-cells at initial diagnosis. Whereas MIP1α exerts potentially suppressive effects on the early myelopoiesis in AA [24,25], Skil functions as negative regulator of TGFβ signaling [25] and its induction may also represent a counterregulatory event.

In comparison to normal controls, marrow-derived CD3+ T-cells showed a large scale of differentially expressed genes indicating that T-cell dysregulation in the BM as target organ is a critical part of the pathological immune response. The regulation of 483 genes also demonstrates that the bone marrow failure results from a rather complex genetic program involving chemokines, cytokines, growth factors, and their receptors. We could identify the induction of several molecules playing key roles in the regulation of Th1 immune responses, such as CCR2 and CX3CR1 [26,27], which are also important in other autoimmune diseases such as multiple sclerosis [28,29], and CD26, a surface-bound ectopeptidase expressed at high levels on Th1 differentiated T-cells [30,31]. The microarray study identified several genes like CD26, PF4 and Ncf1 with distinct immunregulatory functions. As these candidate genes were responding to successful immunotherapy, they might play a key role in the immunpathogenesis of AA and recovery of hematopoiesis after immunosuppression: Specific blocking of CD26, which was upregulated at initial diagnosis and normalized in hematological remission, suppresses the proliferation of autoreactive T-cells with upregulation of TGF-β1 and downregulation of TNF-α [32]; furthermore CD26 favors cytotoxic effector functions in CD8+ T-cells [33]. Specific binding of the chemokine PF4, which was initially enourmosly downregulated and almost normalized after immunotherapy, prevents the release of IL-2 and IFN-γ in activated T-cells and may especially suppress autoreactive T cells in the presence of proinflammatory cytokines [34]. Ncf1, a gene important for oxidative burst formation, regulates the severity of other autoimmune diseases and modulates (in-)directly the level of T-cell dependent autoimmune responses [35]. Taken together, these candidate genes might be highly relevant for the autoreactive T-cell attack in AA and the induction of peripheral tolerance with recovery of the hematopoiesis following immunotherapy.

In contrast to Zeng et al. [19] we could not identify an upregulation of several other chemokines/-receptors (i.e., CCL14, MIP2α, MIP3α, CXCL1 and CXCL12), which play a role in other autoimmune diseases. Also inconsistent with already reported transcript profiles [19] we identifed a downregulation of several molecules involved in innate immune responses, such as Toll-like receptor 2, IL-1 receptor-associated kinase (IRAK3), IL-1 and its receptor in our SAA patient pools. The dysregulation of innate immunity in SAA patients may result in increased proinflammatory cytokine production towards bacterial challenge as observed in IRAK3^-/- ^mice [36]. In accordance to earlier reports [37,38] we could identify a downregulation of IL-1α/β in BM-derived T-cells at initial presentation with normalization of the expression level for IL-1α after hematological recovery. Interestingly, the administration of IL-1 accelerates hematopoietic reconstitution after chemotherapy induced myelosuppression *in vivo *[37] underlining its potential role in marrow failure syndromes. Additionally, the gene expression level of an important regulator of NFκB-dependent proinflammatory signals, CIAS1, was regulated enormously in the pre- and post-therapeutic SAA pools. Interestingly, mutations of CIAS1 cause several autoimmune inflammatory syndromes [39,40] and its downregulation in SAA patients may contribute to the immunpathology of AA by augmentation of proinflammatory signals. As weakly regulated molecules may also have important influence on disease development, we additionally analyzed the gene expression profiles for a minimum fold change of 1.5 and could reveal an upregulation of several genes related to cell-mediated cytotoxicity and apoptosis, such as perforin-1, granzyme H and B [41]. The involvement of cytotoxic granules in the induction of target cell apoptosis has already been shown in AA [42] and other autoimmune disorders [43]. Interestingly, TRAF and TRADD, two mediators of the TNFR familiy for cell activation, survival and antiapoptotic function [44] were upregulated in BM-derived T-cells and may be protective for the effector population in the presence of high TNFα levels.

## Conclusion

In summary, our microarray study revealed a Th1/Tc1 phenotype in bone marrow-derived T-cells of SAA patients at initial presentation. Whereas the potential mechanism for the recruitment of effector T-cell to the bone marrow remained unclear, we could identify several regulators highly relevant for the immunpathogenesis of AA and likely contributing to the recovery of hematopoiesis. Especially the identified regulators of proinflammatory cytokine signaling (IRAK3, CIAS) and molecules with distinct immunregulatory properties in controlling autoreactive T-cells (CD26, PF4, Ncf1) may represent potential targets for the design of new therapeutic strategies.

## Methods

### Patients and controls

Six patients with newly diagnosed severe aplastic anemia (SAA) were included in the study after written informed consent to protocols approved by the Institutional Review Board of the Medizinische Hochschule Hannover. Bone marrow (BM) and peripheral blood (PB) samples were obtained before (n = 6) and after achieving a hematological remission (n = 2) following immunosuppressive therapy with antithymocyte globulin and cyclosporin A (Table 1). The diagnosis of SAA was established by PB counts and BM biopsy as recommended by the International Study of Aplastic Anemia and Agranulocytosis [45]; severitiy was classified according to the criteria of Camitta et al. [46]. Control samples from healthy volunteers were obtained after informed consent according to the institutional guidelines.

### Isolation of CD3^+ ^T cells, RNA preparation and sample pooling

Mononucleated cells of PB and BM were separated by Ficoll-Hypaque sedimentation (Biochrom AG, Berlin, Germany). CD3^+ ^T-cells were positively selected using anti-CD3^+ ^microbeads (Miltenyi-Biotec, Gladbach, Germany) according to the manufacturer's instructions. The purity of the isolated CD3^+ ^T-cells was controlled by FACS analysis and was higher than 96% with comparable CD4^+^/CD8^+ ^ratios. The residual contaminating cell populations varied in the isolations from the different donors and represented natural killer cells (1%–2%), dendritic cells (< 1%), B cells (< 1%), and monocytes (< 1%). Subsequently, total cellular RNA was extracted using TRIzol reagent (Invitrogen, Carlsbad, CA). One μg of total RNA of each preparation, respectively, was pooled seperately for PB and BM as follows: Patients 1–4 prior to therapy (pool I), patients 5 and 6 prior to therapy (pool IIa), patients 5 and 6 after therapy in hematological remission (pool IIb) and healthy donors 1–3 (pool III).

### Affymetrix GeneChip assay

Integrity and quality of total RNA isolated from CD3^+ ^T-cells were checked using Agilent Technologies 2100 Bioanalyser (Agilent Technologies, Germany). To obtain biotinylated cRNA for hybridization a one-cycle target labeling assay was performed. Therefore double-stranded cDNA was synthesized from 2 or 4 μg of pooled total RNA (pools as described above) using T7-oligo(dT)-primers (Eurogentec, Belgium). After clean-up of the ds-cDNA an *in vitro *transcription assay was performed in the presence of biotinylated nucleotides. The amplified and biotinylated cRNA was cleaned up using RNeasy Kit (Qiagen, Germany) and its yield was determined by spectrophotometric analysis. For each GeneChip to be analysed, 10 μg cRNA and additional biotinylated hybridization controls (BioB, BioC, BioD, and Cre) were fragmented and hybridized to an identical lot of HG_U133A GeneChip Arrays (Affymetrix, Santa Clara, CA) for 16 h at 45°C. Afterwards the GeneChips were washed and stained with PE-streptavidin in the Fluidic Station 400 and were read out in the Agilent GeneArray Scanner 2500. All reactions were performed using standard protocols and equipment supplied by the manufacturer.

### Data analysis

Gene expression levels were determined using the Affymetrix Microarray Suite 5.0, MicroDB 3.0 and Affymetrix Data Mining Tool 3.0. For normalization, all array experiments were scaled to a target intensity of 150, otherwise using the default values of the Microarray suite as previously described [47]. Filtering of the results was performed as follows: Genes were considered to be regulated if their fold change was greater/less than or equal to 2. The statistical parameter for a significant change was less than 0.01 [*p*-value for changes called increased (I)] or greater than 0.99 [*p*-value for changes called decreased (D)]. In addition, the signal difference of the respective gene should be greater than 100.

Furthermore, microarray analyses were performed comparing the differential gene expression pattern of SAA patients (pool I and pool IIa of the PB) with profiles of naive and effector memory CD4^+ ^and CD8^+ ^T-cell populations generated from 3 different healthy donors, respectively. For this purpose data available at the EBI Array Express database [13,14] were normalized using the Affymetrix Microarray Suite 5.0 [47]. The normalized data were further analyzed using Affymetrix Assist comparing the expression data of the effector memory T-cell population separately for each donor with the mean gene expression level of the respective naive T-cell populations. Genes were considered to be regulated if their fold change was greater/less than or equal to 2. The statistical parameter for a significant change was p < 0.05 applying the student's t-test.

### Quantitative real-time RT-PCR

Real-time RT-PCR was performed to confirm expression levels of RNA transcripts in pool I, pool IIa, patient 6 after therapy in hematological remission and 2 additional healthy donors. cDNA was synthesized from total RNA using oligo-dT primers and M-MLV polymerase (Invitrogen, Carlsbad, CA) following the manufacturer's recommendations. For relative quantification, RPS9 mRNA served as an external control. The reactions were performed by the ABI PRISM 7000 cycler (Applied Biosystems, Foster City, CA) using SYBR Green PCR Kit (Stratagene, La Jolla, CA) with sequence-specific oligonucleotide primers. A threshold was set in the linear part of the amplification curve, and the number of cycles needed to reach it was calculated for every gene. This threshold cycle was used to quantify mRNA levels of the target genes in the analyzed samples with RPS9 normalization.

## Authors' contributions

AF design of the study, patient sampling, data analysis, writing the ms

RG analysis of the gene expression arrays

JKH performing real-time RT-PCR, data analysis, preperation of tables/figures

SP performing gene expression arrays

WP sample preparation, T-cell isolation

PI data analysis

JG assisting in sample preparation

MPK data analysis

AG design of the study

JB providing array facility, data analysis

## Figures and Tables

**Table 1:** Patients' characteristics.

**patient**	**age/sex**	**diagnosis**	**PNH-clone**	**source of sample**	**pre-therapy sample**	**post-therapy sample**	**pool**
1	19/F	SAA	n	PB BM	y y	n n	I I
2	57/M	vSAA	n	PB BM	y y	n n	I I
3	40/F	SAA	n	PB BM	y y	n n	I I
4	70/F	SAA	n	PB BM	y y	n n	I I
5	64/M	SAA	n	PB BM	y y	y y	IIa/IIb IIa/IIb
6	70/F	vSAA	n	PB BM	y y	y y	IIa/IIb IIa/IIb

Abbreviations: F = female; M = male; (v)SAA = (very) severe aplastic anemia; n = no; y = yes; PB = peripheral blood; BM = bone marrow; I = pool I at initial presentation (n = 4); IIa = pool II at initial presentation (n = 2); IIb = pool II in hematological remission (n = 2). Disease classification was performed according to the International Study of Aplastic Anemia and Agranulocytosis [45].

**Table 2:** Regulation of selected genes expressed in BM-derived T-cells of SAA patients.

**probeset ID**	**Symbol**	**name**	**fold changes vs. control pool**		
			**pool I**	**pool IIa**	**pool IIb**
207075_at	CIAS1	cold autoinflammatory syndrome 1	- 23.49	- 6.29	- 2.12
210118_s_at	IL-1 A	interleukin-1, alpha	- 25.90	- 40.28	- 3.49
220034_at	IRAK3	interleukin-1 receptor-associated kinase 3	- 5.91	- 88.03	- 2.18
204961_s_at	NCF1	neutrophil cytosolic factor 1	- 3.87	- 2.23	- 1.32
206390_x_at	PF4	platelet factor 4	- 156.82	- 187.53	- 11.81
211743_s_at	PRG2	proteoglycan 2	- 236.22	- 78.96	- 2.55
204924_at	TLR2	toll-like receptor 2	- 10.43	- 5.04	- 2.22

Abbreviations: I = pool I at initial presentation (n = 4); IIa = pool II at initial presentation (n = 2); IIb = pool II in hematological remission (n = 2).
